# Physico-Chemical Properties and Biocompatibility of Thermosensitive Chitosan Lactate and Chitosan Chloride Hydrogels Developed for Tissue Engineering Application

**DOI:** 10.3390/jfb12020037

**Published:** 2021-05-20

**Authors:** Katarzyna Pieklarz, Grzegorz Galita, Michał Tylman, Waldemar Maniukiewicz, Ewa Kucharska, Ireneusz Majsterek, Zofia Modrzejewska

**Affiliations:** 1 Department of Environmental Engineering, Faculty of Process and Environmental Engineering, Lodz University of Technology, Wolczanska 213 Street, 90-924 Lodz, Poland; zofia.modrzejewska@p.lodz.pl; 2 Department of Clinical Chemistry and Biochemistry, Medical University of Lodz, Narutowicza 60 Street, 90-136 Lodz, Poland; grzegorz.galita@umed.lodz.pl (G.G.); ireneusz.majsterek@umed.lodz.pl (I.M.); 3 PGE Gornictwo i Energetyka Konwencjonalna S.A., Weglowa 5 Street, 97-400 Belchatow, Poland; michal.tylman1@gmail.com; 4 Institute of General and Ecological Chemistry, Faculty of Chemistry, Lodz University of Technology, Zeromskiego 116 Street, 90-924 Lodz, Poland; waldemar.maniukiewicz@p.lodz.pl; 5 Department of Gerontology, Geriatrics and Social Work, Jesuit University Ignatianum in Krakow, Kopernika 26 Street, 31-501 Krakow, Poland; ewa.kucharska@ignatianum.edu.pl

**Keywords:** tissue engineering, natural polymer, chitosan, thermosensitive hydrogel, structural properties, cytotoxicity, genotoxicity

## Abstract

Recently, the modification of the initial structure of biopolymers, mainly chitosan, has been gaining importance with a view to obtain functional forms with increased practicality and specific properties enabling their use in tissue engineering. Therefore, in this article, the properties (structural and biological) of thermosensitive hydrogels obtained from chitosan lactate/chloride and two types of crosslinking agents (β-glycerol phosphate disodium salt pentahydrate and uridine 5′-monophosphate disodium salt) are discussed. The aim of the research is to identify changes in the structure of the biomaterials during conditioning in water. Structural investigations were carried out by FTIR spectroscopy. The crystallinity of gels was determined by X-ray diffraction analysis. The biocompatibility (evaluation of cytotoxicity and genotoxicity) of chitosan hydrogels was investigated by contact with human colon adenocarcinoma cell line for 48 h. The cytotoxicity was verified based on the colorimetric resazurin assay, and the genotoxicity was checked by the comet assay (percentage of DNA in the comet tail). The conducted research showed that the analyzed types of chitosan hydrogels are non-cytotoxic and non-genotoxic materials. The good biocompatibility of chitosan hydrogels surfaces makes them interesting scaffolds with clinical potential in tissue regeneration engineering.

## 1. Introduction

For several years, the research undertaken in the field of tissue engineering, which is an innovative but intensively developing discipline of science based on the issues related to the fields of materials science, biology, biotechnology, chemistry, and biochemistry, has been gaining importance [1,2,3]. This is because the standard methods of treating damaged tissues, such as pharmacotherapy and transplant techniques, are often of limited effectiveness. Therefore, the solutions offered by tissue engineering constitute an alternative. They do not require the use of materials with a low degree of biocompatibility and postpone the need for arthroplasty, allowing the patient to return to full mobility and to their everyday activities [4,5].

The essence of tissue engineering is to create biomaterials that can replace or regenerate damaged tissue and restore its basic function. The regeneration process takes place in several stages. The first step is the biopsy of a piece of tissue that is processed to isolate the necessary cells from the extracellular matrix. The next component of the process is to multiply the cells (in vitro) and seed them on a scaffold, and then cultivate them in a bioreactor under optimal environmental conditions, culture medium and growth factors. The final step is the implementation (in vivo) of the obtained scaffold with multiplied cells into the patient’s body. It is also important that the material for the scaffold is slowly degraded and resorbed [6,7,8,9].

Currently, the manufacture of new materials for scaffolds is a trend in global scientific research. Hydrogels, which are three-dimensional hydrophilic matrices made of crosslinked homopolymers, copolymers or macromers, occupy a special position [10]. Given the nature of crosslinks, there are physical and chemical hydrogels. The first type of gels is created by various reversible links, for example van der Waals interactions, hydrogen bonds or electrostatic interactions. These interactions can be induced and reversed depending on the pH or the temperature making them especially useful in biomedicine. In turn, the chemical systems are formed by irreversible covalent links and compared with physically crosslinked gels are more stable under physiological conditions and have better mechanical properties [11,12,13].

Due to the relatively high water content, softness and plasticity, hydrogels exhibit similar physical properties to living tissues. These biomaterials, apart from being used in tissue engineering, are an excellent material for obtaining immunoisolation barriers for microencapsulation technology and dressings for the treatment of skin damage and burns. Additionally, they are applied as smart carriers in controlled drug delivery systems. This solution ensures a safe and effective therapy. Unlike conventional drug delivery systems in which the substance is released immediately after entering the body, the drug takes effect after a certain period and is dosed evenly. Moreover, hydrogels are used as gene carriers and as integral components of microdevices such as biosensors [14,15,16,17,18,19].

An extremely interesting form of hydrogels are temperature-sensitive systems, which can undergo a phase transition at the human body temperature. In general, they consist of hydrophobic and hydrophilic components, and the thermal response is caused by the equilibrium between the above parts of the polymer monomer [20].

The main advantage of thermosensitive hydrogels is the possibility of their noninvasive introduction into the pathologically affected area by injection, which avoids first-pass metabolism. In addition, these biomaterials can assume a shape that perfectly matches the tissue damage. This removes the need for a surgical operation, reduces the adhesion problems of cells and bioactive substances, and eliminates the difficulties associated with their even dispersion in the structure. This is due to the possibility of introducing cells and therapeutic agents into the solution before administration—in situ [20,21,22].

Hydrogels with thermosensitive properties are mainly obtained from poly (N-isopropylacrylamide)-based (PNIPAAM) copolymers, poly (ethylene glycol)/biodegradable polyester copolymers, chitosan and its derivatives [23,24].

**Figure 1:**
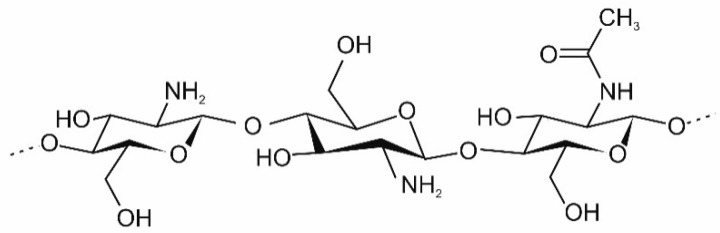
Structure of chitosan.

Among the above polymers, chitosan (Figure 1), a polysaccharide resulting from the alkaline deacetylation of chitin, is a particularly promising material for scaffolds. This biopolymer exhibits many unique biological, physiological, and pharmacological properties: nontoxicity, biocompatibility with living tissues and biodegradability. The bioactivity of chitosan is also noteworthy, including acceleration of the wound healing process, increased immunity, hemostatic activity, induction of a biological response, bactericidal and fungicidal activity [25,26,27,28].

Chitosan is degraded in the human body by several physiological enzymes (lysozyme, di-N-acetylchitobiase, N-acetyl-beta-d glucosaminidase and chitiotriosidase etc.), and the process of its biodegradation causes release of nontoxic oligosaccharides [29,30,31,32].

However, it should be borne in mind that the biocompatibility of this material can be influenced many factors, for example the degree of deacetylation, particle size, concentration, or route of administration [33]. There are also studies pointing to the potential of chitosan and its degradation products to activate human macrophages and lymphocyte proliferation without symptoms of inflammation [32,34].

The current legislation states that each new material intended to be used in the biomedical field must be subject to extensive research aimed at assessing its cytotoxicity before referring it to clinical trials. In addition, each modification of a previously assessed solution may affect its potential cytotoxic activity.

**Figure 2:**
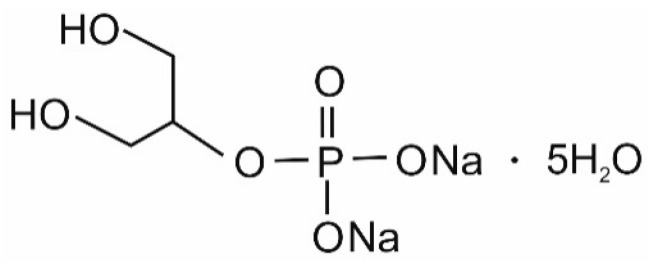
Structure of β-glycerol phosphate disodium salt pentahydrate.

**Figure 3:**
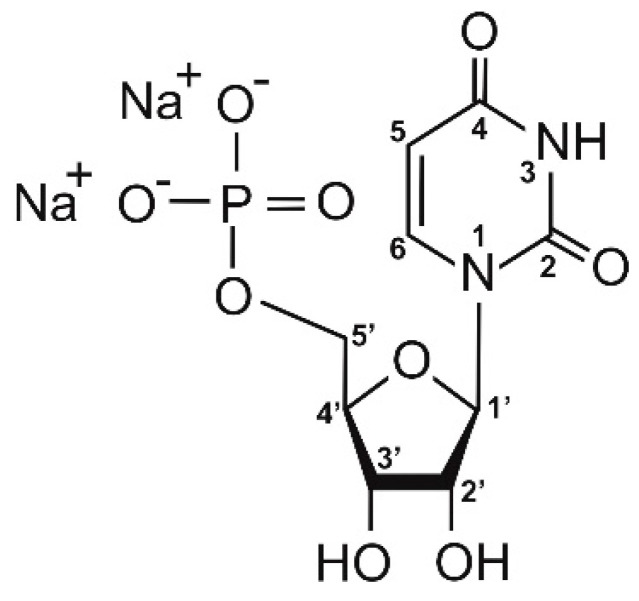
Structure of uridine 5′-monophosphate disodium salt.

In this paper, hydrogels with thermosensitive properties are shown. These systems were formed from chitosan lactate and chitosan chloride solutions with the use of β-glycerol phosphate disodium salt pentahydrate (β-GP) (Figure 2) and uridine 5′-monophosphate disodium salt (UMP) (Figure 3) as the crosslinking agents.

Uridine 5′-monophosphate disodium salt is an organic chemical compound, a nucleotide that is a major component of ribonucleic acid. This neuroactive molecule plays an important function in the pyrimidine metabolism of the brain, furnishes nervous tissue with the pyrimidine ring and takes part in the metabolic pathways. In addition, UMP acts as a signaling molecule in the central nervous system and participates in the control of physiological and pathophysiological brain functions. This nucleotide can be used in the treatment of neurodegenerative diseases and polyneuropathy and in the therapy of the myelin sheath lesion [35,36].

The literature suggests that chitosan hydrogels formed at 37 °C are most obtained by the addition of β-glycerol phosphate disodium salt pentahydrate [37,38,39,40,41].

However, there is no information on the preparation of the thermo-gelling systems with the participation of uridine 5′-monophosphate disodium salt. Only the production of biomaterials using derivatives of uridine (oUrd) and uridine monophosphate (oUMP) in combination with glutaraldehyde (AG) has been discussed, but these systems did not show phase transition under the influence of the temperature [42].

A review of the available publications presents that the hydrogels with thermosensitive properties formed by the presence of uridine 5′-monophosphate disodium salt is only found in our previous article [43]. The study was carried out to determine the structural properties of pure hydrogels, prepared from chitosan lactate and chitosan chloride, based on FTIR spectra analysis and morphology of these biomaterials evaluated by scanning electron microscope (SEM). In addition, to assess the state of water in the structure of hydrogels, thermal analysis was performed using a differential scanning calorimetry (DSC).

In turn, this publication is focused on the determination of changes in the structure of the gels containing β-GP and UMP due to conditioning in water, which is particularly important when considering the possibility of using hydrogels, for example as scaffolds in tissue engineering or carriers for controlled drug release.

The obtained biomaterials were characterized by Fourier transform infrared spectroscopy (FTIR) and X-ray diffraction (XRD) studies.

In turn, the biological research included the evaluation of the cytotoxicity and genotoxicity of the developed chitosan hydrogels using human colon adenocarcinoma cell line (HT-29 cell line).

## 2. Materials and Methods

### 2.1. Materials of Hydrogels

Chitosan (CH) product no. 50494-100GF, lactic acid (LA) product no. L6661-100ML, hydrochloric acid (HCL) product no. H1758-100ML, the crosslinking agents: β-glycerol phosphate disodium salt pentahydrate (β-GP) product no. 50020-100G and uridine 5′-monophosphate disodium salt (UMP) product no. U6375-10G were purchased from Sigma-Aldrich (Poznan, Poland). Deionized water treated by a water purification system (Elga Purelab, High Wycombe, UK) was used in the preparation of chitosan hydrogels and their conditioning. All chemical reagents were of analytical grade and were used without further purification.

### 2.2. Preparation of Solutions and Hydrogels Manufacture

**Figure 4:**
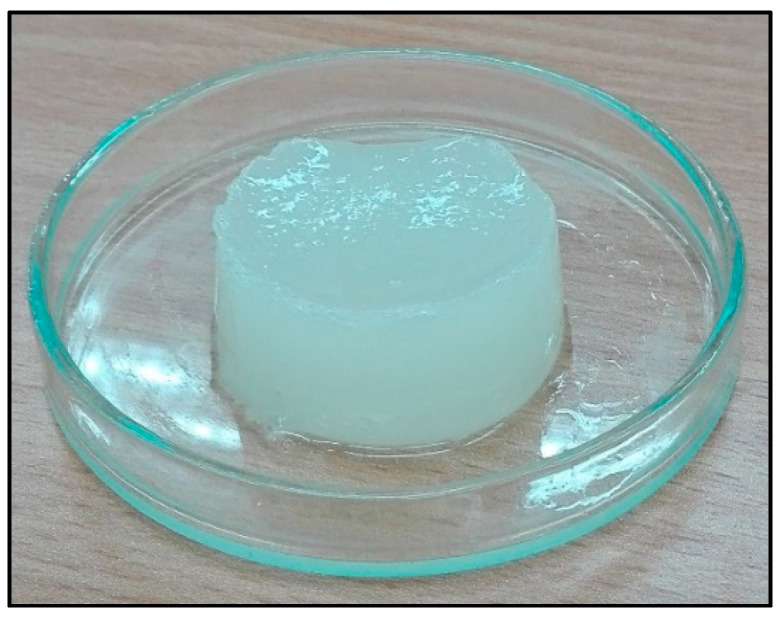
Thermosensitive chitosan hydrogel (the CH/LA/UMP system).

Thermosensitive chitosan hydrogels were prepared by physical blending. Firstly, CH (0.4 g) was dissolved in 16 mL LA or HCL (0.1 mol/L). The solutions were stirred (at slow rotations) until complete dissolution and left at room temperature for 24 h. Then, solutions of the crosslinking agents (2 g β-GP was dissolved in 2 mL deionized water at 4 °C, while 2 g UMP was dissolved in 2.5 mL deionized water at 4 °C) were added drop by drop into the chitosan salts solutions. Each sample was mixed for 20 min and stored at 4 °C for about 1 h. The prepared formulations were homogeneous solutions, which were subsequently incubated at 37 °C to complete their gelation. Due to the fact that the obtained hydrogels looked the same, only a photograph of the CH/LA/UMP system is shown in Figure 4.

### 2.3. In Vitro Conditioning

The prepared thermosensitive chitosan hydrogels were subjected to the in vitro release process carried out under static conditions without mixing. The release process of β-GP and UMP was studied in 100 mL deionized water, maintaining a constant at 37 °C.

### 2.4. Physico-Chemical Studies

The structural properties of chitosan gels before and after conditioning in water for 1, 2, 4, 6, 8, 24, 48 and 72 h were analyzed. Each of the samples for analysis were frozen at −20 °C and then lyophilized under the pressure of 0.63 mbar and the temperature of −25 °C for about 48 h using the Martin Christ Freeze Dryer ALPHA 2-4.

#### 2.4.1. Fourier Transform Infrared Spectroscopy

Fourier transform infrared (FTIR) spectra of the lyophilized chitosan hydrogels were characterized using a Nicolet™ iS50 FT-IR apparatus equipped with a monolithic diamond ATR crystal (Thermo Fisher Scientific Inc., Madison, WI, USA). All spectra were recorded with 100 scans at a 4.0 cm^−1^ resolution in the range of wavenumbers 4000–500 cm^−1^.

#### 2.4.2. X-ray Diffraction

The crystalline structure of obtained hydrogels was accessed by the room temperature powder X-ray diffraction technique. The study was performed in the PANanalytical X’Pert Pro MPD diffractometer in the Bragg–Brentano reflection geometry with (CuKα) radiation from a sealed tube (Malvern Panalytical Ltd., Royston, UK). The Cu radiation was generated at 30 mA and 40 kV. The apparatus operated in the range of 2θ = 3–40°, with a step size of 0.0167°, and the measuring time was 20 s/step.

### 2.5. Biological Studies

#### 2.5.1. Cell Culture

The analysis of biological properties of the chitosan biomaterials was carried out on the commercially available human colon adenocarcinoma cell line (HT-29 cell line) purchased from the American Type Culture Collection (ATCC; Manassas, VA, USA). The cells were cultured in McCoy’s 5A medium (Sigma-Aldrich Corp., St. Louis, MO, USA) supplemented with 10% (*v*/*v*) fetal bovine serum (FBS; Sigma-Aldrich Corp., St. Louis, MO, USA), 100 units/mL penicillin and 100 μg/mL streptomycin (both from GIBCO-BRL, Life Technologies Ltd., Paisley, Scotland). After exposure to accutase solution, the cells were passaged at 85–95% confluency.

#### 2.5.2. Preparation of CH Solutions for Cytotoxicity and Genotoxicity Studies

Sterile CH formulations for the biological studies were prepared under aseptic conditions in a vertical laminar airflow cabinet equipped with UV sterilization and HEPA filters (PCR Workstation by Labcaire Systems Ltd., Clevedon, UK).

#### 2.5.3. Cytotoxicity Analysis

To evaluate the cytotoxic effect of the hydrogels on the studied cell line, the colorimetric resazurin assay, In Vitro Toxicology Assay Kit, Resazurin based (Sigma-Aldrich Corp., St. Louis, MO, USA) was performed. All analyses were carried out in triplicate with similar results. HT-29 cells were seeded in 12-well plates (2 × 10^5^ cells/well) and cultured in 1 mL of complete McCoy’s 5A medium for 24 h. The cells suspended in 1 mL of complete culture medium were used as a negative control, whereas cells treated with 100% DMSO (Sigma-Aldrich Corp., St. Louis, MO, USA) as a positive control. After cells’ adhesion, the cells were incubated with small pieces of hydrogels (diameter: 5 mm) for 48 h. Following incubation, the well contents were removed, and the cells were rinsed twice with 1 X DPBS. Subsequently, 100 μL of the resazurin solution was added to each well, and the cells were incubated for 2 h. Absorbance was measured at a wavelength of 600 nm and a reference wavelength of 690 nm using Synergy HT (BioTek) spectrophotometer.

#### 2.5.4. Genotoxicity Assessment

The genotoxicity of the analyzed hydrogels was estimated using the alkaline version of the comet assay. HT-29 cells were seeded in 12-well plates (2 × 10^5^ cells/well) and cultured in 1 mL of complete McCoy’s 5A medium for 24 h. The cells suspended in 1 mL of complete culture medium were used as a negative control, whereas cells treated with 10% DMSO as a positive control. After cells’ adhesion, the cells were incubated with small pieces of hydrogels (diameter: 5 mm) for 48 h. Following incubation, the well contents were removed, and 0.2 mL of accutase/well was added to harvest the cells. The harvested cells were centrifuged. Cell suspension in 0.37% LMP agarose (Sigma-Aldrich Corp., St. Louis, MO, USA) was placed on microscope slides, which were previously coated with NMP agarose (Sigma-Aldrich Corp., St. Louis, MO, USA). Subsequently, the preparations were incubated in lysis buffer (2.5-M NaCl, 10-mM Tris, 100-mM EDTA, pH 10) with the addition of 1% TritonX-100 (Sigma-Aldrich Corp., St. Louis, MO, USA) at 4 °C for 1 h. Following the lysis, the preparations were incubated in development buffer (300 mM NaOH, 1 mM EDTA) at 4 °C for 20 min, followed by electrophoresis (17 V, 32 mA, 20 min) at 4 °C in an electrophoretic buffer (30 mM NaOH, 1 mM EDTA). Then, the slides were rinsed with distilled water and left to dry completely. The obtained preparations were stained with a DAPI fluorescent dye and examined under a fluorescent microscope to assess the level of DNA damage.

#### 2.5.5. Statistical Analysis

Statistical analysis was carried out using a nonparametric technique: the Mann–Whitney test in statistical program Sigma Plot (Systat Software, Inc.). Each of the analyses in individual experiments were based on the results of three independent tests. Significant statistical differences were presented as follows: * *p* < 0.05, ** *p* < 0.01, *** *p* < 0.001 versus the negative control.

## 3. Results and Discussion

### 3.1. Fourier Transform Infrared (FTIR) Spectra

To identify individual functional groups, FTIR spectra of chitosan hydrogels were made. The structure of the biomaterials formed with the use of β-glycerol phosphate disodium salt pentahydrate and uridine 5′-monophosphate disodium salt was compared.

**Figure 5:**
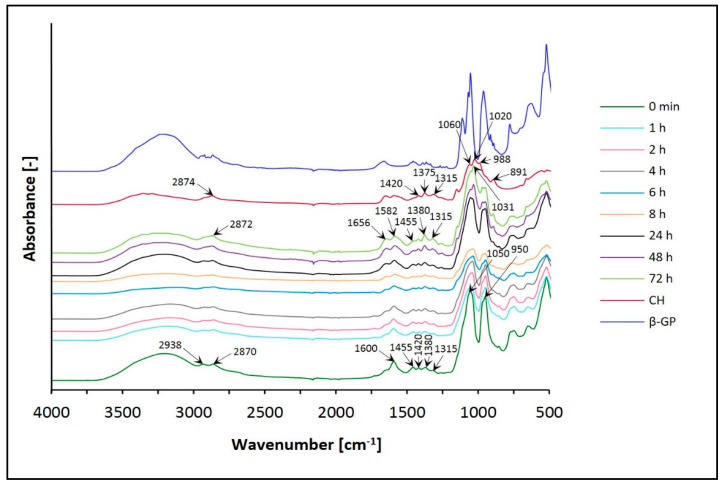
FTIR spectra of the CH/LA/β-GP system conditioned in water.

**Figure 6:**
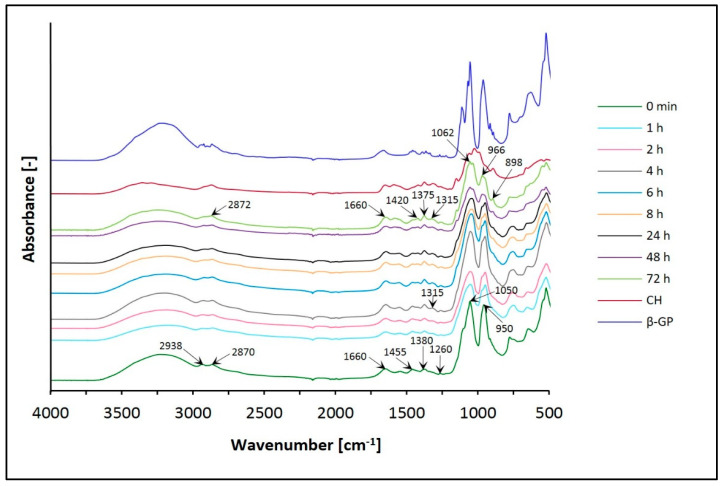
FTIR spectra of the CH/HCL/β-GP system conditioned in water.

The results in Figure 5 and Figure 6 show the spectra of chitosan lactate hydrogels (the CH/LA/β-GP system) and chitosan chloride (the CH/HCL/β-GP system) with β-glycerol phosphate disodium salt pentahydrate after different times of conditioning in water and, for comparison, the spectra of the gels before conditioning (0 min) and the spectrum of chitosan (CH) powder.

**Figure 7:**
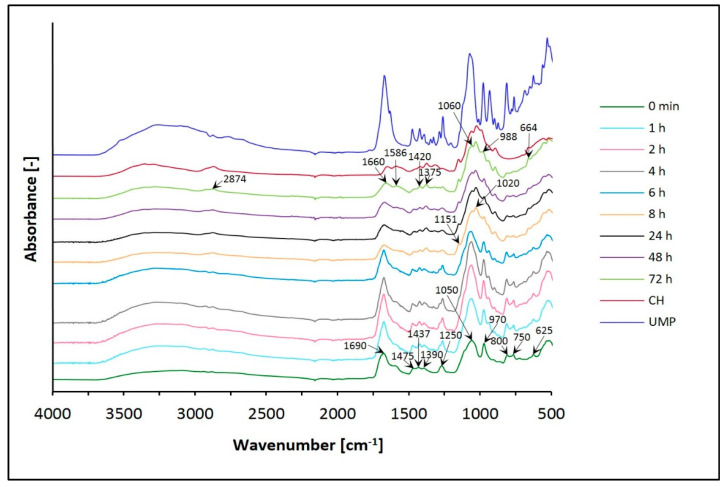
FTIR spectra of the CH/LA/UMP system conditioned in water.

**Figure 8:**
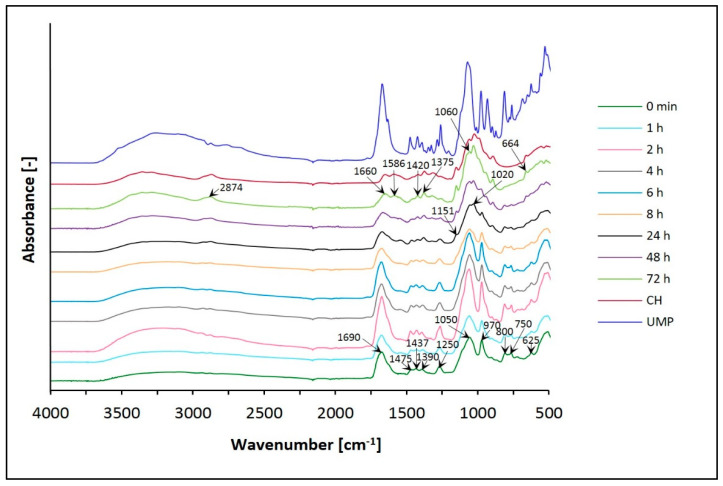
FTIR spectra of the CH/HCL/UMP system conditioned in water.

On the other hand, Figure 7 and Figure 8 present the spectra of chitosan lactate hydrogels (the CH/LA/UMP system) and chitosan chloride (the CH/HCL/UMP system) with uridine 5′-monophosphate disodium salt.

In the spectra of chitosan hydrogels with β-glycerol phosphate disodium salt pentahydrate (the CH/LA/β-GP and the CH/HCL/β-GP systems), the broad, asymmetric spectrum in the range of wavenumbers 3600–3100 cm^−1^ is assigned in all cases before and after conditioning in water. This band corresponds to the O–H stretching, indicating intermolecular hydrogen bonding which overlaps the asymmetric stretching vibrations of NH_2_ groups and the N–H stretching vibrations between molecules N–H…O=C in the same region. For hydrogels before conditioning (0 min), the band is shifted towards lower wavenumbers compared to the spectrum of CH, and after conditioning this band moves to higher frequencies and becomes more symmetric.

In the range of 2950–2850 cm^−1^, the spectrum of CH has an asymmetric band at 2874 cm^−1^, which consists of two overlapping bands representing the –CH_2_ and –CH_3_ aliphatic groups, characteristic of the pyranose ring of CH. The spectra of both types of hydrogels (0 min) are split into two distinct bands at 2938 and 2870 cm^−1^. In the case of biomaterials conditioned in water, this band is observed at 2872 cm^−1^ and has a minor shoulder.

Analyzing the frequency range of 1680–1500 cm^−1^, it is possible to observe characteristic peaks of CH, corresponding to the C=O bond in amide group (amide I vibration) and the amide II band coming from NH_2_, which indicate that this polymer is a partially deacetylated product of chitin. The spectra of the CH/LA/β-GP system (0 min) and hydrogels after conditioning in water for less than 8 h show one distinct band of 1600 cm^−1^ with a minor shoulder. Biomaterials conditioned in water for above 8 h have two bands at 1656 and 1582 cm^−1^. On the other hand, in the case of the CH/HCL/β-GP systems (before and after conditioning in water), no significant changes are observed.

In the range of wavenumbers 1500–1200 cm^−1^, the spectrum of CH has four peaks associated with oscillations characteristic of the O–H and C–H bending of CH_2_ groups and representing the C–O stretching of the primary alcoholic group –CH_2_–OH (1420, 1375, 1315 and 1260 cm^−1^). For all the CH/LA/β-GP system variants (before and after conditioning in water), bands are observed at 1420, 1315 and 1260 cm^−1^, as in the case of CH. Additionally, a peak appears at 1455 cm^−1^, and the band at 1375 cm^−1^ is shifted to 1380 cm^−1^. In turn, the spectra of the CH/HCL/β-GP systems (0 min and conditioned in water for less than 4 h) present three peaks at 1455, 1380 and 1260 cm^−1^. For biomaterials conditioned in water for 4 and more hours, there is an additional band at 1315 cm^−1^, as in the case of CH. For hydrogels conditioned for 6 to less than 72 h, the spectrum shows five peaks at 1455, 1420, 1380, 1315 and 1260 cm^−1^. After conditioning for 72 h, only bands typical of the CH molecule are observed.

The spectrum of CH in the range of 1200–800 cm^−1^ shows bands at 1151, 1060, 1020, 988 and 891 cm^−1^, characteristic of saccharide structure (oxygen bridge bond (C–O–C) and CH_3_COH groups). Before conditioning in water, the CH/LA/β-GP system does not have the bands typical for a CH molecule. However, two new bands, at 1050 and 950 cm^−1^ with a minor shoulder at 980 cm^−1^, connected with the presence of β-glycerol phosphate disodium salt pentahydrate appear in this region. The band at 1050 cm^−1^ indicates the aliphatic P–O–C stretching, the band at 980 cm^−1^ is characteristic of the –PO_4_^2–^ group, whereas the band at 950 cm^−1^ may indicate the presence of the –HPO_4_^–^ group. In the case of the CH/HCL/β-GP system (0 min), a band at 1050 cm^−1^ with a small arm for the wavenumber 1100 cm^−1^ and a peak at 950 cm^−1^ is observed. The spectra for the CH/LA/β-GP systems conditioned in water for more than 24 h have peaks typical of CH (1148, 1031 and 894 cm^−1^). For the CH/HCL/β-GP systems conditioned for longer than 24 h, there are four bands at 1062, 1031, 966 and 898 cm^−1^. The bands resulting from the presence of phosphorus in the hydrogels structure disappear.

The spectrum of CH in the range of 800–500 cm^−1^ has one band at 664 cm^−1^, which relates to the vibrations of the O=C–N group. For both types of hydrogels, before conditioning in water, there are bands at 750, 650 and 523 cm^−1^. The band at 750 cm^−1^ is characteristic of β-GP (the aliphatic P–O–C stretching). In this frequency range, no significant changes are observed for all samples conditioned in water.

Interpretation of the FTIR spectra was based on previous studies [44,45,46,47,48,49].

The spectra of hydrogels with UMP vary with time, as do the spectra of systems with β-GP. Changes, in the broad band range 3600–3000 cm^−1^ and for the peak around 2850 cm^−1^ but primarily in the region of 1750–600 cm^−1^ are observed.

After conditioning in water, the band in the range of wavenumbers 3600–3000 cm^−1^ moves toward higher frequencies. In the spectrum of CH, two maxima (3360 and 3295 cm^−1^) can be observed; in both types of hydrogels, the O–H and N–H bands overlap.

Moreover, spectra obtained for both variants of biomaterials conditioned in water show the band at 2874 cm^−1^, which is typical of the CH molecule (–CH_2_ and –CH_3_ aliphatic groups).

Analyzing the frequency range of 1750–600 cm^−^^1^, major changes are observed, which is related to the presence of uridine 5′-monophosphate disodium salt in the hydrogels structure.

Before conditioning in water, in the FTIR spectra of the CH/LA/UMP and the CH/HCL/UMP systems, characteristic peaks are detected at wavenumbers: around 1690 cm^−1^ (C(2)=O stretching mode), near 1475 cm^−1^ (in plane deformation mode of N(3) –H), 1437 cm^−1^ (deformation mode of O–H [ribose]), 1390 cm^−1^ (deformation mode of O–H [ribose] and in plane deformation mode of N(3) –H), around 1250 cm^−1^ (ring stretching mode of [N(1) –C(2) –N(3)] and C–H bending mode in uracil), 1050 cm^−1^ (C–C stretching mode in ribose, C–O stretching mode in ribose, N(1) –C(1′) stretching mode), 970 cm^−1^ (symmetrical stretching mode of PO_3_^2–^), 900 cm^−1^ (C–C stretching mode in ribose), 800 cm^−1^ (P–O stretching mode, C–C stretching mode in ribose and C–H rocking mode in uracil), 750 cm^−1^ (C–H rocking mode in uracil and C=O rocking mode) and 625 cm^−1^ (C–C–O bending mode in ribose and C=O bending mode).

After conditioning in water, for both types of hydrogels, the band at 1690 cm^−1^ becomes sharper at first, but from 8 h of conditioning it is less intense and moves towards lower frequencies. After a sufficiently long time (72 h), it appears at a wavenumber similar to that in the CH spectrum. Additionally, a peak appears at 1586 cm^−1^ for both systems after 72 h. On the other hand, the band at 1475 cm^−1^ is present but with a little more intensity. After conditioning in water, the next two peaks at 1437 and 1390 cm^−1^ move towards lower wavenumbers and appear at 1420 and 1375 cm^−1^, respectively, as for the CH molecule. In the case of the band around 1250 cm^−1^, intensity decreases for both types of biomaterials conditioned for longer than 8 h.

In the range of wavenumbers 1200–800 cm^−1^, in the spectrum of the CH/LA/UMP system from 8 h of conditioning, and the CH/HCL/UMP system from 24 h, bands appear at 1151 and 1020 cm^−1^; they are characteristic of the saccharide structure. The peaks at the frequencies of 1050 and 970 cm^−1^, along with the extension of the conditioning time, are less intense for both systems and shift towards higher wavelengths: 1060 and 988 cm^−1^, respectively, as in the spectrum of pure chitosan. Moreover, in the spectra of both types of hydrogels conditioned in water, the band at 900 cm^−1^ does not have significant changes.

In turn, the bands at 800 and 750 cm^−1^ are clearly visible up to a defined time (24 h) and then they disappear leaving trace amounts. The peak at 625 cm^−1^, in the spectra of the CH/LA/UMP and the CH/HCL/UMP systems, moves towards a higher wavenumber (664 cm^−1^).

Interpretation of the FTIR spectra was based on previous studies [50,51,52].

### 3.2. Crystallinity—XRD Diffractograms

The structural changes of the hydrogels were defined based on powder X-ray diffraction analysis (XRD).

**Figure 9:**
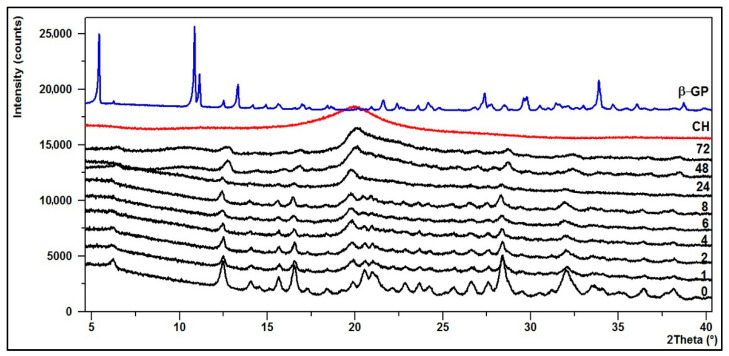
XRD diffraction patterns of the CH/LA/β-GP system conditioned in water.

**Figure 10:**
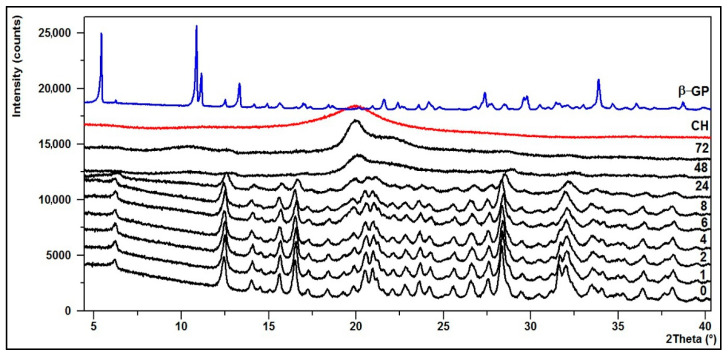
XRD diffraction patterns of the CH/HCL/β-GP system conditioned in water.

The diffraction patterns of the CH/LA/β-GP and the CH/HCL/β-GP systems after conditioning in water are shown in Figure 9 and Figure 10.

**Figure 11:**
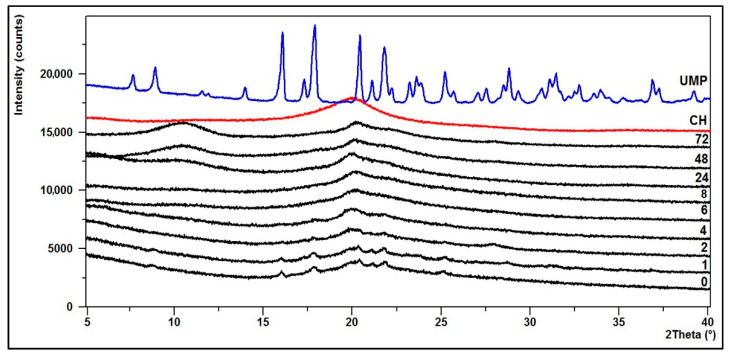
XRD diffraction patterns of the CH/LA/UMP system conditioned in water.

**Figure 12:**
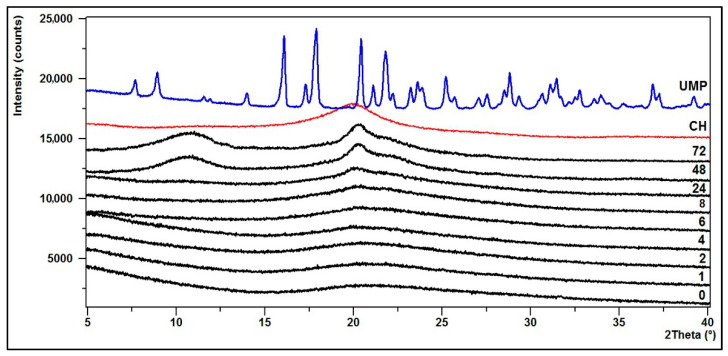
XRD diffraction patterns of the CH/HCL/UMP system conditioned in water.

In turn, Figure 11 and Figure 12 present the diffraction patterns of chitosan lactate hydrogels (the CH/LA/UMP system) and chitosan chloride (the CH/HCL/UMP system) with uridine 5′-monophosphate disodium salt.

The XRD pattern of the starting polymer (CH) is characterized by a typical reflex at 2θ ≈ 20°, which indicates that chitosan as a polymer with low crystallinity index is a rather amorphous body [53].

Upon the transformation of chitosan into thermosensitive hydrogels, its structure changes. The diffractogram of the CH/LA/β-GP system (0 min) shows that the hydrogel is partially crystalline and characterized by a number of small bands and five distinct peaks with maxima at 2θ angles of about 12, 17, 20, 28 and 32°. Similar features are visible on the diffractogram of the CH/HCL/β-GP system (0 min), but in this case the peaks are more intense and stronger. This is indicative of a structure with a higher crystallinity due to the formation of a compound between glycerophosphate and chitosan or precipitation of sodium chloride, which appeared after drying.

In the case of hydrogels conditioned in water for up to 8 (the CH/LA/β-GP system) or 24 h (the CH/HCL/β-GP system), their structure does not significantly change although the intensity of the peaks decreases. However, after longer conditioning, due to the leaching of sodium glycerophosphate, the structure of gels returns to pure chitosan.

The diffractogram of the CH/LA/UMP system (0 min) shows that the hydrogel is partially crystalline and characterized by six peaks at the angles of 2θ = 16, 18, 20.5, 21, 22 and 25°. In the case of the hydrogel conditioned in water for up to 4 h, the intensity of the above peaks decreases. After 6 h, the XRD pattern starts to resemble the diffractogram of a pure polymer. From 48 h of conditioning, two bands at the angles of 2θ ≈ 11 and 20°, characteristic for the CH molecule, are clearly visible.

On the other hand, the diffractogram for the CH/HCL/UMP system (0 min) shows that the biomaterial is practically amorphous. From 24 h of conditioning in water, the structure of the hydrogel returns to pure polymer and from 48 h, two typical peaks at angles of 2θ ≈ 11 and 20° are noticeable.

The presented diffractograms confirm the suggestions resulting from the analysis of FTIR spectra.

### 3.3. Analysis of the Cytotoxicity of Chitosan Hydrogels

Although chitosan hydrogels crosslinked by β-GP are highly studied in biomedical research, no information is available as to the biocompatibility of systems with UMP. Therefore, the safety profile of both types of gels was compared in the present work.

The resazurin assay, a quantitative and rapid colorimetric method, was chosen to preliminarily screen the cytotoxicity range of the CH/LA/β-GP, CH/HCL/β-GP, CH/LA/UMP and CH/HCL/UMP systems. This method is based on the reduction of oxidized non-fluorescent blue resazurin to a pink, fluorescent dye (resorufin) by cell activity (likely by oxygen consumption through metabolism) [54,55].

**Figure 13:**
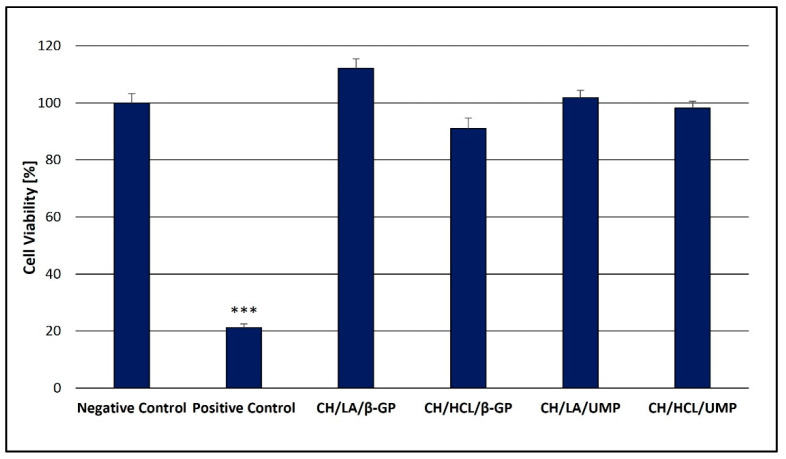
Cytotoxicity of the chitosan hydrogels. *** *p* < 0.001 versus negative control.

The results shown in Figure 13 summarize the viability of HT-29 cells incubated with the hydrogels for 48 h.

The studies on cytotoxicity revealed that in the case of all the tested hydrogels, irrespective of the solvent and crosslinking agent used, no negative impact on human colon adenocarcinoma cells (HT-29) was observed.

In the case of chitosan lactate gels (the CH/LA/β-GP and the CH/LA/UMP systems), increased cell proliferation in relation to the negative control is observed (median of cell viability: 112.1 and 101.8% respectively). Slightly higher cytotoxic response is noticed for biomaterials prepared from chitosan chloride (the CH/HCL/β-GP and the CH/HCL/UMP systems)—the median of cell viability is 91.0 and 98.2%, respectively—which is probably due to the fact that hydrochloric acid has a lower pH (pH < 1) than lactic acid (pH 2.44).

### 3.4. Evaluation of Genotoxicity of Chitosan Hydrogels

Apart from cytotoxicity assessment, the evaluation of genotoxicity is another factor determining the use of biomaterials in medicine. The advantage of in vitro studies on biocompatibility is that multiple samples can be evaluated simultaneously. Moreover, only the materials that appear to be effective can be further analyzed in vivo in experimental models. The use of cell culture assays allows for a quick and easy examination of cellular processes using small amounts of the tested substance, and their great advantage is repeatability. These tests are ethically more acceptable in comparison to in vivo animal experiments and, more importantly, their results may lead to significant clinical conclusions in biomaterial research [56].

In this study, a genotoxicity assessment of the chitosan hydrogels was performed using the alkaline version of the comet assay. This technique is a highly specific, sensitive, and rapid method, which enables the detection of oxidative DNA damage, single- and double-stranded breaks as well as the presence of alkaline labile sites. The first versions of the test were developed in 1978 by Rydberg and Johanson, who described the detection of single-strand DNA breaks in individual cells embedded in agarose [57,58].

**Figure 14:**
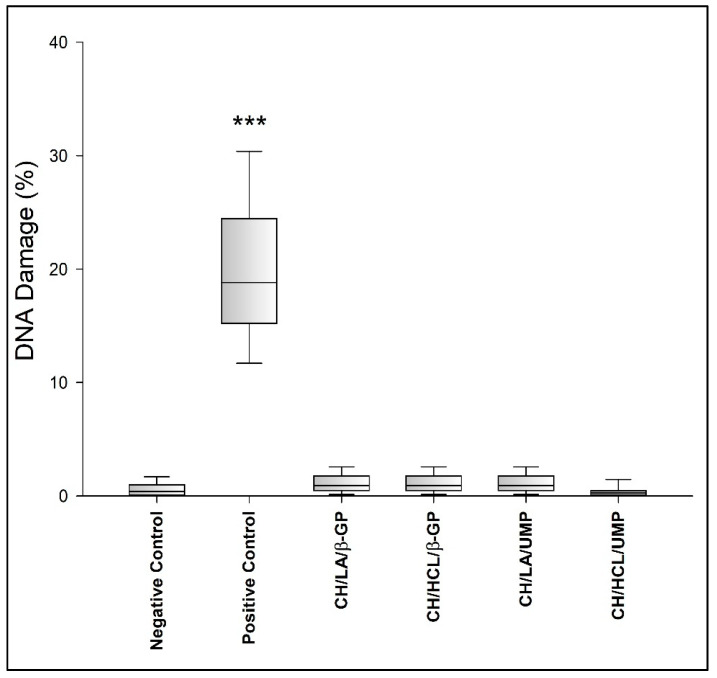
Genotoxicity of the chitosan hydrogels. *** *p* < 0.001 versus negative control.

**Figure 15:**
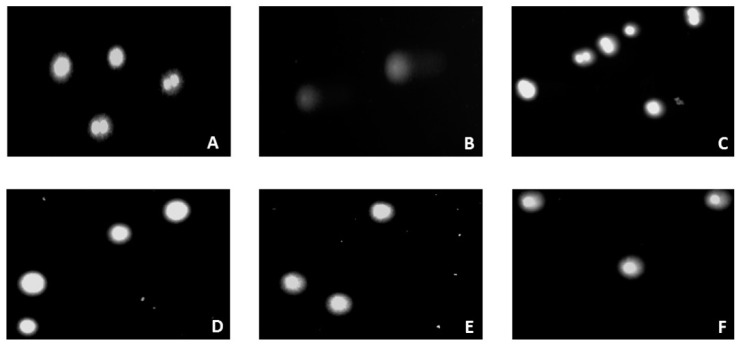
Representative images (200 × magnification) of the comet assay in HT-29 cells after 48 h of exposure: (**A**) negative control, (**B**) positive control, (**C**) CH/LA/β-GP treated, (**D**) CH/HCL/β-GP treated, (**E**) CH/LA/UMP treated, (**F**) CH/HCL/UMP treated.

The amount of DNA damage was assessed based on the percentage of DNA in the comet tail (Figure 14 and Figure 15).

The obtained results show that all tested variants of chitosan hydrogels, irrespective of the solvent and crosslinking agent used, do not induce significant DNA damage in HT-29 cells (all systems give <2% DNA damage). As depicted in Figure 15, cells exposed to chitosan hydrogels predominantly display a round-shaped head, which is part of the undamaged DNA without visible tail (fragmented DNA) similar to the negative control cells (A), indicating that the hydrogels and their components do not induce a genotoxic effect in human colon adenocarcinoma cells.

The findings of the biological study suggested that all the analyzed chitosan hydrogels could be candidates for scaffolds with good biocompatibility. However, due to the fact that the potential area of application of the developed biomaterials, in particular the hydrogels containing UMP, is neural tissue engineering, further studies using, for example, the astrocyte cell line (C8-D1A line), should be carried out.

## 4. Conclusions

This study demonstrates the thermosensitive chitosan lactate and chitosan chloride hydrogels. The biomaterials were prepared via sol–gel technique with the use of two crosslinking agents (β-glycerol phosphate disodium salt pentahydrate (β-GP) and uridine 5′-monophosphate disodium salt (UMP)).

Based on structural studies, it was found that the structure of all analyzed hydrogels changed due to conditioning in water. Changes in FTIR spectra of hydrogels with β-GP can be observed in the range of the broad band 3600–3100 cm^−1^, but mainly in the range of the amide band 1680–1500 cm^−1^, band 1500–1200 cm^−1^ and band 1200–800 cm^−1^, which reflects the saccharide structure and is connected with the presence of phosphate ions. In the case of biomaterials with UMP, the most significant changes are recorded in the frequency range of 1750–600 cm^−1^. Regardless of the solvent and type of crosslinking agent used, conditioning of the hydrogels in water leads to FTIR spectra corresponding to the spectrum of the pure polymer. The analysis of spectra showed that the biomaterials containing UMP are characterized by faster release of the crosslinking agent than the gels with β-GP. Thus, the advantage of UMP could be its presence for delivering it in a possible neuronal growth procedure, which requires additional studies to be performed on the retention/release of UMP from the gel in the future. The obtained results of the analysis carried out with the room temperature powder X-ray diffraction technique confirm the general conclusions resulting from the FTIR spectra.

Since the anticipated field of application of the manufactured hydrogels is biomedicine, a crucial factor is their safety for the human body. Therefore, an important step was to assess the biocompatibility of the hydrogels in contact with the human colon adenocarcinoma cell line. Biological studies showed that biomaterials are non-cytotoxic and non-genotoxic, and the chitosan lactate gels (the CH/LA/β-GP and the CH/LA/UMP systems) even increase the cell proliferation in relation to the negative control.

Thus, the obtained hydrogels can be proposed as scaffolds for potential application in the clinical and tissue engineering field, being a promising tool in tissue-constructs development, for example nervous tissue, due to the application of the pyrimidine ribonucleotide (UMP), which has a regenerative effect on the components of the nervous system by improving neurotransmission.

## 5. Patents

Majsterek I., Modrzejewska Z., Pieklarz K., Tylman M.; Method for producing chitosan gels forming in the human body temperature, intended for injection scaffolds for breeding of nerve cells. Lodz University of Technology, Lodz. Poland. Patent application 235369. Publ. 29.06.2020 WUP.

## Data Availability

Data sharing is not applicable to this article.
